# Loss of chromosome 10 is an independent prognostic factor in high-grade gliomas

**DOI:** 10.1038/sj.bjc.6693403

**Published:** 1999-12

**Authors:** S Balesaria, C Brock, M Bower, J Clark, S K Nicholson, P Lewis, S de Sanctis, H Evans, D Peterson, N Mendoza, M G Glaser, E S Newlands, R A Fisher

**Affiliations:** Department of Cancer Medicine, Imperial College School of Medicine, Charing Cross Hospital, Fulham Palace Road, London W6 8RF, UK; Department of Oncology, Chelsea & Westminster Hospital, London, UK; Division of Neuroscience, Imperial College School of Medicine; Department of Histopathology andDepartment of Neurosurgery, Charing Cross Hospital, London, UK

**Keywords:** glioma, brain tumour, loss of heterozygosity, microsatellite

## Abstract

Loss of heterozygosity (LOH) for chromosome 10 is the most frequent genetic abnormality observed in high-grade gliomas. We have used fluorescent microsatellite markers to examine a series of 83 patients, 34 with anaplastic astrocytoma (grade 3) and 49 with glioblastoma multiforme (grade 4), for LOH of chromosome 10. Genotype analysis revealed LOH for all informative chromosome 10 markers in 12 (35%) of patients with grade 3 and 29 (59%) grade 4 tumours respectively, while partial LOH was found in a further eight (24%) grade 3 and ten (20%) grade 4 tumours. Partial LOH, was confined to the long arm (10q) in six and the short arm (10p) in three cases, while alleles from both arms were lost in four cases. Five tumours (one grade 3 and four grade 4) showed heterogeneity with respect to loss at different loci. There was a correlation between any chromosome 10 loss and poorer performance status at presentation (χ^2^*P* = 0.005) and with increasing age at diagnosis (Mann–Whitney *U*-test *P* = 0.034) but not with tumour grade (χ^2^*P* = 0.051). A Cox multivariate model for survival duration identified age (proportional hazards (PH), *P* = 0.004), grade (PH, *P* = 0.012) and any loss of chromosome 10 (PH, *P* = 0.009) as the only independent prognostic variables. Specifically, LOH for chromosome 10 was able to identify a subgroup of patients with grade 3 tumours who had a significantly shorter survival time. We conclude that LOH for chromosome 10 is an independent, adverse prognostic variable in high-grade glioma. © 1999 Cancer Research Campaign


*British Journal of Cancer* (1999) **81**(8), 1371–1377
© 1999 Cancer Research Campaign
Article no. bjoc.1999.0854

### Loss of chromosome 10 is an independent prognostic factor in high-grade gliomas

**S Balesaria[1], C Brock[1], M Bower[2], J Clark[3], SK Nicholson[1], P Lewis[4], S de Sanctis[4], H Evans[1], D Peterson[5], N Mendoza[5], MG Glaser[1], ES Newlands[1] and RA Fisher[1]**

[1]Department of Cancer Medicine, Imperial College School of Medicine, Charing Cross Hospital, Fulham Palace Road, London W6 8RF, UK; [2]Department of Oncology, Chelsea & Westminster Hospital, London, UK; [3]Division of Neuroscience, Imperial College School of Medicine, [4]Department of Histopathology and [5]Department of Neurosurgery, Charing Cross Hospital, London, UK

**Summary** Loss of heterozygosity (LOH) for chromosome 10 is the most frequent genetic abnormality observed in high-grade gliomas. We have used fluorescent microsatellite markers to examine a series of 83 patients, 34 with anaplastic astrocytoma (grade 3) and 49 with glioblastoma multiforme (grade 4), for LOH of chromosome 10. Genotype analysis revealed LOH for all informative chromosome 10 markers in 12 (35%) of patients with grade 3 and 29 (59%) grade 4 tumours respectively, while partial LOH was found in a further eight (24%) grade 3 and ten (20%) grade 4 tumours. Partial LOH, was confined to the long arm (10q) in six and the short arm (10p) in three cases, while alleles from both arms were lost in four cases. Five tumours (one grade 3 and four grade 4) showed heterogeneity with respect to loss at different loci. There was a correlation between any chromosome 10 loss and poorer performance status at presentation ($\chi^2$ $P$ = 0.005) and with increasing age at diagnosis (Mann–Whitney *U*-test $P$ = 0.034) but not with tumour grade ($\chi^2$ $P$ = 0.051). A Cox multivariate model for survival duration identified age (proportional hazards (PH), $P$ = 0.004), grade (PH, $P$ = 0.012) and any loss of chromosome 10 (PH, $P$ = 0.009) as the only independent prognostic variables. Specifically, LOH for chromosome 10 was able to identify a subgroup of patients with grade 3 tumours who had a significantly shorter survival time. We conclude that LOH for chromosome 10 is an independent, adverse prognostic variable in high-grade glioma.

© 1999 Cancer Research Campaign

**Keywords:** glioma; brain tumour; loss of heterozygosity; microsatellite

Gliomas are the most frequently occurring central nervous system (CNS) tumours and include astrocytomas, anaplastic astrocytomas and glioblastoma multiforme. They are severely disabling, producing symptoms of headaches, fits, confusion, personality changes and neurological deficits which impair the patients' quality of life. Despite advances in the diagnosis and treatment of gliomas the prognosis remains poor, with only 10–15% survival for patients with high-grade gliomas at 30+ months (Brada et al, 1998). Nevertheless, considerable variation exists with respect to post-operative survival. Identification of prognostic variables to assist in clinical management is thus of considerable value to both patients and clinicians.

Historically, prognostic models for gliomas have used clinical variables and patient characteristics to determine factors that predict response to therapy and survival. In 1990, the Medical Research Council (MRC) working party developed a clinical scoring system that predicted a better prognosis for patients less than 45 years old at diagnosis, with longer duration of fits, debulking surgery and a better performance status (Report of the MRC Brain Tumour Working Party, 1990). Although adverse histological features including high proliferative activity (Bruner, 1994), the presence of necrosis (Barker et al, 1996) and a gemistocyte population of greater than 20% (Krouer et al, 1991) have been identified in univariate analysis of small series, these have not been evaluated in larger series and are not widely employed.

The development of gliomas, in common with many tumours, is a multistep process involving the accumulation of several genetic events which involve the activation of oncogenes (Collins, 1995) and the loss of tumour suppressor genes (Jen et al, 1994; Louis and Gusella, 1995; von Deimling et al, 1995). The commonest genetic abnormality observed in gliomas is loss, often involving a whole homologue, of chromosome 10 (Bigner at al, 1990). This loss is associated with high-grade tumours, being rare in low-grade astrocytomas but present in some 60–85% of high-grade gliomas (Louis and Gusella, 1995). Recent studies have led to the identification of at least three tumour suppressor genes, PTEN (MMAC1), DMBT1 and LGI1 (Li et al, 1997; Mollenhauser et al, 1997; Steck et al, 1997; Chernova et al, 1998) on chromosome 10, which may be involved in glioma progression. The aim of this study was to determine whether loss of heterozygosity (LOH) for chromosome 10 is an important prognostic variable in high-grade gliomas and whether it provides additional predictive information to the previously defined clinical prognostic indices.

### MATERIALS AND METHODS

### Patients

Between January 1994 and April 1997, blood samples and pathological blocks of tumour material were collected prospectively from 42 patients diagnosed with high-grade glioma. Blood tumour

*Received 15 December 1998*
*Revised 15 March 1999*
*Accepted 25 May 1999*

*Correspondence to:* RA Fisher pairs were obtained from a further 44 patients with high-grade glioma who were being followed up in the oncology department and for whom tumour blocks were available from the biopsy at initial diagnosis.

### Pathological diagnosis

Stained sections of tumour were examined by two independent pathologists and tumours classified on the basis of criteria described by Daumas-Duport et al (1998) including the presence or absence of nuclear atypia, mitoses, endothelial proliferation and necrosis. Where two of the first three criteria were present, almost always nuclear atypia and mitosis, the tumours were categorized as grade 3 or anaplastic astrocytomas (AA). If all three criteria were present, the tumour was categorized as grade 4 or glioblastoma multiforme (GBM). Any tumour with necrosis, in addition to one or more of the other three criteria, was classified as grade 4. Of the 86 tumours examined, 36 were diagnosed as AA (grade 3) and 50 as GBM (grade 4). Two cases of AA and one case of GBM were subsequently excluded because DNA prepared from the pathological blocks failed to amplify reproducibly.

### Clinical variables

Clinical details including the age at diagnosis, gender, duration of fits and nature of initial surgery were recorded for all patients. Eastern Co-operative Oncology Group (ECOG) performance status at diagnosis was prospectively recorded for 39 of 42 newly diagnosed patients and was available for 35 of the 41 remaining patients.

### LOH for chromosome 10

DNA was prepared from blood samples using standard techniques. Tumour tissue for DNA preparation was obtained from pathological blocks of tumour removed at the time of initial surgery. Microdissecton of glioma tissue from an unstained section was performed by comparison with a consecutive section stained with haematoxylin and eosin to distinguish areas of tumour from any normal brain tissue. DNA was then prepared using a modification of the method described by Wright and Manos (1990) (Fisher et al 1997).

A total of 50 ng of DNA, prepared from the patients blood and 1 μl tumour DNA were amplified using a control pair of primers for one or more microsatellites on unrelated chromosomes, in order to assess the quality of the tumour DNA. Blood–tumour pairs were then amplified using a panel of six pairs of primers which flank microsatellite repeat sequences on chromosome 10 (Figure 1). To facilitate analysis, one of each pair of primers was labelled with the fluorescent dye FAM (blue) or HEX (green).

Polymerase chain reaction (PCR) products were subsequently resolved either by electrophoresis in 6% denaturing polyacrylamide gels using an ABI 373A automated DNA sequencer (Applied Biosystems Ltd, Cheshire, UK) or capillary electrophoresis using an ABI Prism 310 Genetic Analyser. Using ABI 672 or ABI Prism GeneScan software (Applied Biosystems Ltd), sizes of fluorescent bands, representing different alleles, were automatically determined by comparison with an internal size standard. Where microsatellite polymorphisms were heterozygous in the blood, the alleles present in the tumour were examined for LOH. A small number of tumours were found to show allelic imbalance rather than complete loss of one allele. In these cases, the ratios of the height of the two alleles was calculated for the blood (N1/N2) and tumour (T1/T2). Allelic imbalance was calculated from the ratio of tumour signal to that of the normal signal (T1/T2 over N1/N2). Ratios of $< 0.67$ or $> 1.35$ were taken to indicate LOH for that locus.

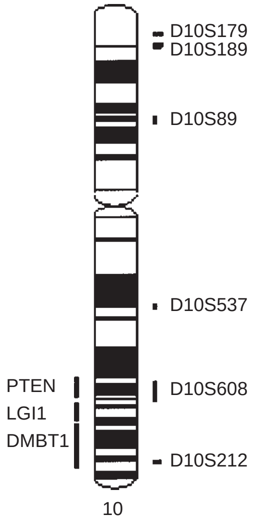

**Figure 1** Cytogenetic localization of microsatellite markers used to identify LOH for chromosome 10 (GDB, 1998). The location of the tumour suppressor genes, PTEN (MMAC1) (Li et al, 1997; Steck et al, 1997), DBMT1 (Mollenhauser et al, 1997) and LGI1 (Chernova et al, 1998) are shown in relation to the markers examined

### Statistical methods

Survival was calculated from the day of diagnosis until death or the date of last follow-up. Overall survival duration curves were plotted according to the method of Kaplan and Meier (1958). The log-rank method was used to test for the significance of differences in survival distributions (Peto et al, 1977). Multivariate analysis of significant variables was performed using a stepwise Cox regression model to establish which were independently prognostic (Cox, 1972).

### RESULTS

### Clinicopathological details

Tumour samples have been analysed from 83 patients with high-grade gliomas including 34 AA and 49 GBM. The median age of patients at presentation was 49 years (range 17–78) and 50 were male (60%). The performance status at presentation was recorded prospectively for 73 patients and was ECOG grade 0 for six patients (8%), grade 1 for 16 (22%), grade 2 for 30 (41%) and grade 3 for 21 (29%). Initial surgery was biopsy alone for 46 (55%) and debulking surgery for 37 (45%). Sixty-nine patients (83%) had whole brain irradiation following initial surgery. Forty-three patients (51%) also received chemotherapy either as adjuvant therapy or at relapse, including 38 who were enrolled on trials of temozolomide (Newlands et al, 1996).

 © 1999 Cancer Research Campaign

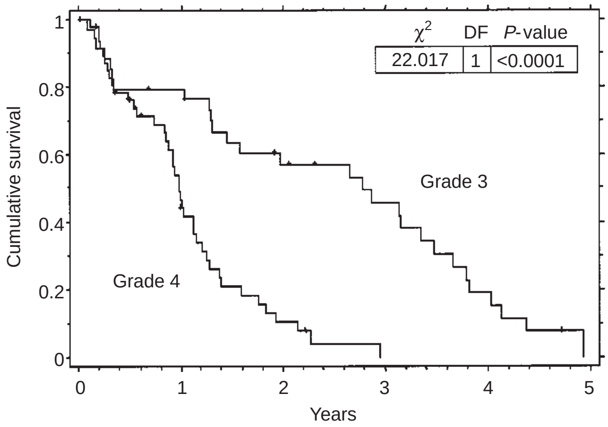

**Figure 2** Kaplan–Meier overall survival duration curves according to tumour grade

### Treatment outcome

The median overall survival from diagnosis is 1.24 years and the 1- and 2-year actuarial survival rates for the cohort are 63% (95% confidence interval (CI) 52–74%) and 32% (95% CI 21–43%) respectively. Patients with grade 3 tumours survived longer than those with grade 4 tumours (log-rank, $P$ < 0.0001) (Figure 2). Patients who underwent debulking surgery lived no longer than those who had only a biopsy (log-rank, $P$ = 0.34). A better performance status at presentation was also associated with a trend towards longer survival although this failed to reach statistical significance (log-rank, $P$ = 0.056). There was no difference in survival between the genders (log-rank, $P$ = 0.50) or in relation to duration of fits prior to diagnosis (proportional hazards, $P$ = 0.39). Younger patients survived longer (proportional hazards, $P$ < 0.0001).

### LOH for chromosome 10

Genotype analysis revealed no loss (Figure 3A) for any of the loci examined on chromosome 10 in 24 (29%) tumours. Of the remaining 59 tumours, 41 (49% of all cases) demonstrated LOH (Figure 3B) for all chromosome 10 loci tested, while 18 (22% of all cases) had partial LOH, i.e. LOH for only some informative loci. Partial LOH was confined to the long arm (10q) in six and the short arm (10p) in three cases, whilst alleles from both arms were lost in four cases (Figure 4). The five remaining tumours showed heterogeneity with respect to LOH, in that some markers showed a greater degree of loss within the tumour than others. This suggests that for these five tumours, at least, two or more different cell populations were present within the sample. Complete LOH for chromosome 10 was observed more frequently in grade 4 (59%) than grade 3 tumours (35%) while partial loss occurred at a higher frequency in grade 3 (24%) than grade 4 (20%). No loss was more frequently associated with grade 3 (38%) than grade 4 (22%) tumours.

### Prognostic factors

Within high grade tumours, any loss of chromosome 10 was associated with a reduced overall survival (log-rank, $P$ = 0.0001)

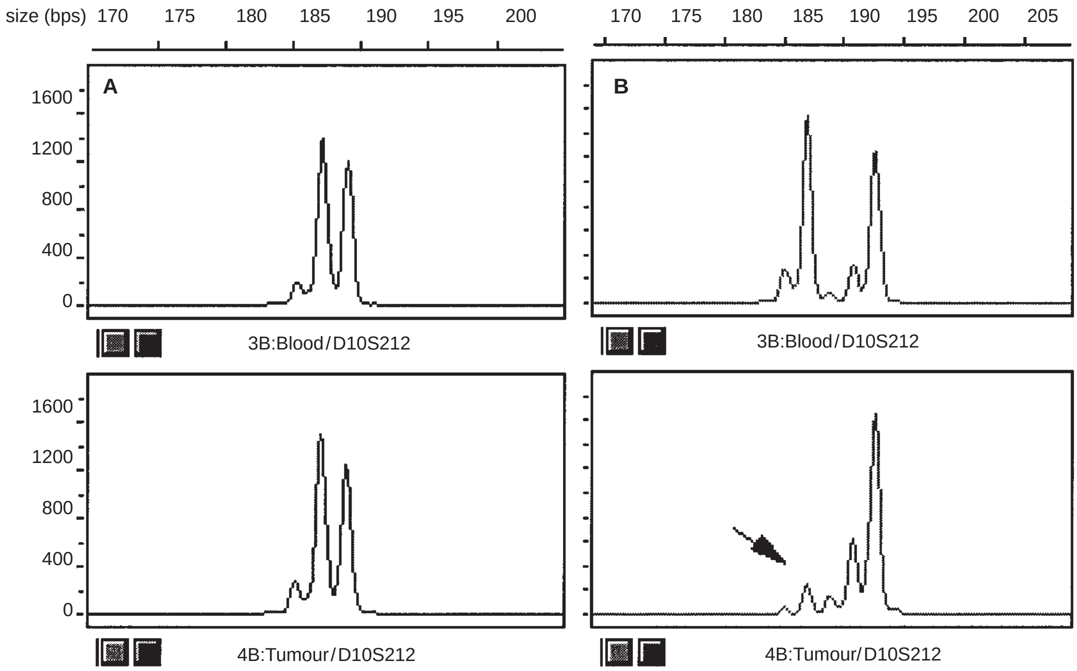

**Figure 3** Microsatellite polymorphisms detected following PCR amplification with primers for the dinucleotide repeats D10S212 in cases gli 44 and gli 4 respectively. Fluorescently labelled microsatellites are displayed as characteristic electropherogram peaks following gel electrophoresis. Sizes of alleles, in base pairs (bps), are those calculated using GeneScan software. Variation of dinucleotide peak heights within samples represents preferential amplification of smaller alleles. (**A**) The relative height of the two alleles in the tumour DNA is not significantly different to that in DNA from the blood, indicating no LOH for this locus in the tumour. (**B**) DNA from the blood sample is heterozygous for the D10S212 microsatellite. However, the tumour DNA has only a single allele indicating LOH (arrowed) for this locus

*© 1999 Cancer Research Campaign*

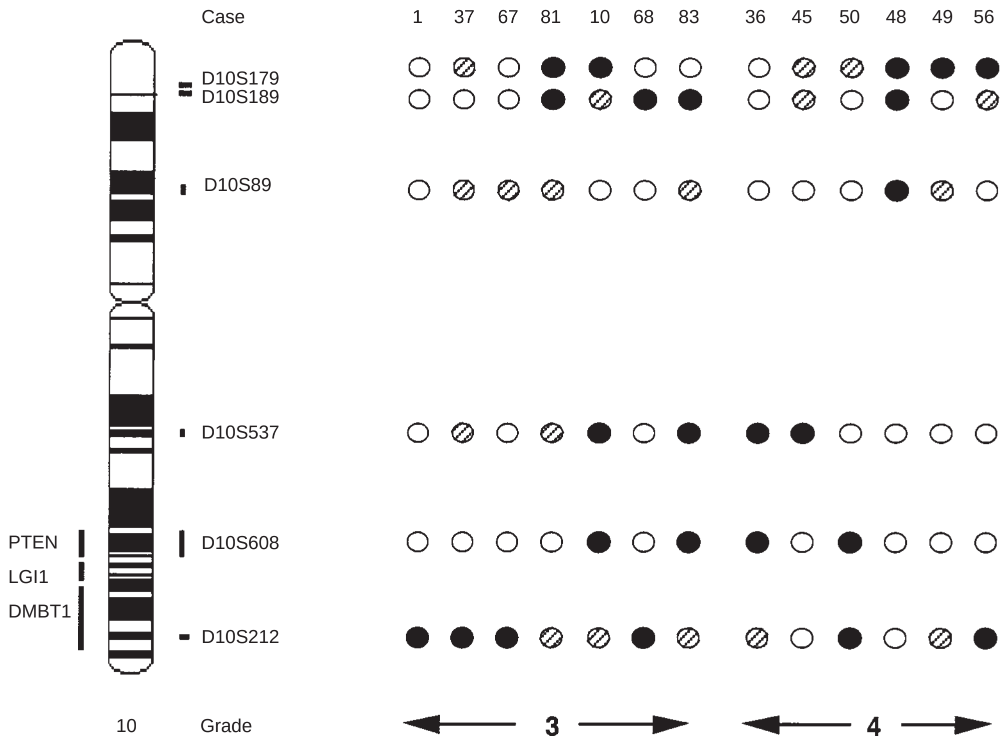

**Figure 4** Thirteen cases in which partial LOH was identified. LOH for a locus is represented by (●) no LOH by (○) and uninformative markers by (◍)

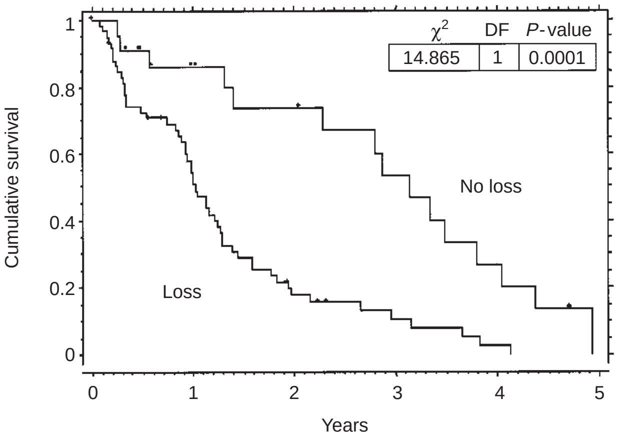

**Figure 5** Kaplan–Meier overall survival duration curves for all patients according to chromosome 10 status of the tumour

(Figure 5). Median survival for patients with complete or partial LOH for chromosome 10 was 0.99 years, while median survival for patients without LOH for 10 was 2.9 years. There was a correlation between any chromosome 10 loss and poorer performance status at presentation ($\chi^2$, $P = 0.005$), increasing age at diagnosis (Mann-Whitney *U*-test, $P = 0.029$) and with tumour grade ($\chi^2$, $P = 0.04$) but not initial surgery ($\chi^2$, $P = 0.40$). A Cox multivariate model for survival duration identified age (hazard ratio (HR) = 1.04, 95% CI 1.02–1.06, $P = 0.004$), grade (HR = 0.34, 95% CI 0.18–0.65, $P = 0.001$) and any loss of chromosome 10 (HR = 1.87, 95% CI 1.00–3.60, $P = 0.050$) as the only independent prognostic variables.

When patients with grade 3 or grade 4 tumours were considered independently, patients who had grade 3 tumours with LOH for chromosome 10 showed a significantly reduced overall survival (log-rank, $P = 0.012$) (Figure 6A), median survival for the two groups being 1.5 years and 3.5 years respectively. For patients with grade 4 tumours, with LOH or without LOH for chromosome 10, the median survival was 0.95 and 1.20 years respectively. Although patients with grade 4 tumours with LOH showed shorter overall survival, this did not achieve significance (log-rank, $P = 0.26$) (Figure 6B).

### DISCUSSION

The most frequently occurring central nervous system (CNS) tumours, the astrocytic gliomas, have a very poor prognosis; high-grade gliomas being almost universally fatal despite surgery, radiotherapy and chemotherapy. Within this group of patients a number of clinical and pathological variables have been examined with respect to prognosis, the most significant prognostic variables in other series being age at diagnosis, extent of surgery, clinical performance status at time of diagnosis (Report of the MRC Brain Tumour Working Party, 1990) and tumour grade (Chang et al, 1993).

In our series the extent of initial surgery did not affect survival. While a better performance status at presentation was associated with longer survival this did not reach significance. Multivariate analysis confirmed age to be an independent prognostic variable, in that younger patients survived longer. Similarly, tumour grade was shown to be an independent prognostic variable, patients with grade 4 tumours surviving for a significantly shorter period than those with grade 3 tumours. The treatment offered to the cohort

© 1999 Cancer Research Campaign

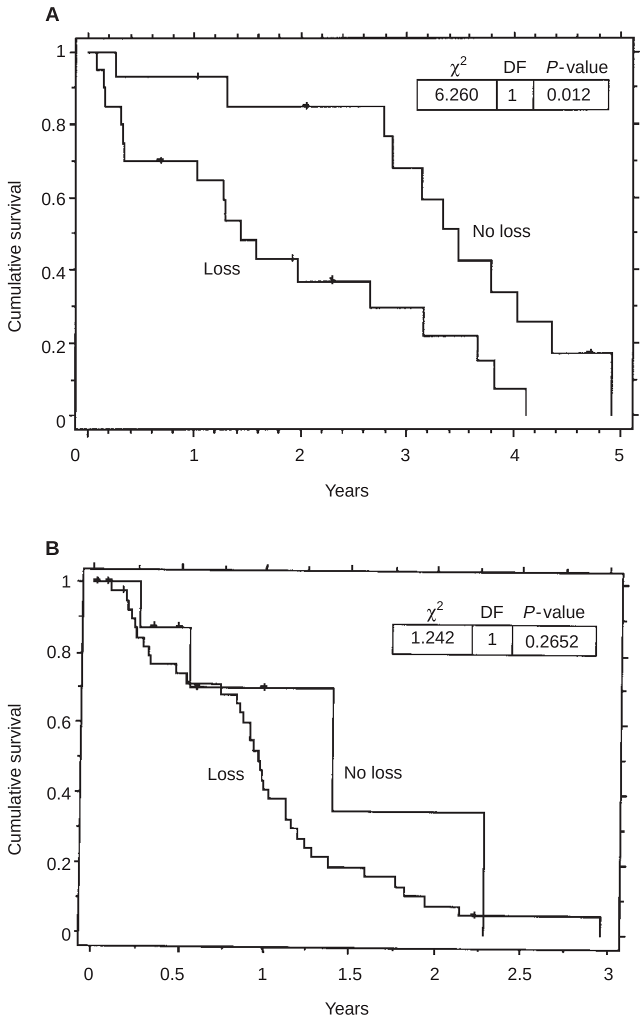

**Figure 6** Kaplan–Meier overall survival duration curves according to chromosome 10 status of the tumour in patients with (**A**) grade 3 and (**B**) grade 4 tumours following initial surgery varied and both radiotherapy and chemotherapy schedules have altered over the course of recruitment. Therefore the influence of post-surgical treatments cannot be reliably evaluated. It has been shown that adjuvant treatment following surgery has little influence on median survival in patients with high grade glioma (Brada et al, 1998). Variation in treatment is therefore unlikely to significantly influence prognosis.

It has recently been suggested that some of the genetic changes, associated with glioma development and progression, may have prognostic significance. While amplification and loss of specific regions of the genome have been found in a high proportion of gliomas, not all tumours have been shown to have the same combination of genetic alterations. This variation may reflect distinct molecular pathways of tumour progression (Louis and Gusella, 1995; von Deimling et al, 1995) and represent subgroups of tumours with different prognosis.

Alterations of p53, frequently associated with tumours which show progression through low- to high-grade and amplification of the epidermal growth factor receptor (EGFR), associated with high grade glioma which arise 'de novo', are two of the most commonly observed genetic changes in glioma. However, the prognostic significance of overexpression of the EGFR (Waha et al, 1996; Zhu et al, 1996; Rainov et al, 1997) and mutations or aberrant expression of p53 (Campomenosi et al, 1996; Cunningham et al, 1997; Korkolopoulou et al, 1997), remains equivocal.

The most frequently observed genetic alteration in high-grade tumours is LOH for chromosome 10. Despite this a significant proportion of high-grade gliomas show no LOH for chromosome 10 markers. Poor prognosis has been associated with abnormalities of chromosome 10 (Ganju et al, 1994), while patients with p53 gene alteration in their tumour were found to have a significantly shorter survival time if in addition they had complete LOH for chromosome 10 (Leenstra et al, 1998). Our aim was to determine whether LOH for chromosome 10 was in fact an independent prognostic variable. The present study includes 83 patients with high-grade tumours of whom 69 (83%) have been followed up until death. Patients with either complete or partial loss of chromosome 10 showed a significantly reduced survival time compared to those patients with no LOH for chromosome 10. Multivariate analysis showed that this loss was independent of other prognostic variables considered. When patients with grade 3 or grade 4 tumours were considered independently, grade 4 tumour cases with LOH showed lower overall survival but this did not achieve significance. This may reflect the relatively small number of cases in this group which did not show any LOH. In patients with grade 3 tumours, LOH was associated with a significantly reduced overall survival. Analysis of chromosome 10 loss is thus able to identify a subgroup of patients with anaplastic astrocytomas who have a very poor prognosis.

In common with other reports, most cases in our study with LOH for chromosome 10, involved loss of the whole homologue. Frequent loss of the whole homologue suggests the involvement of several different tumour suppressor genes on chromosome 10 in glioma development. This is confirmed by the observation that, where partial loss is found, it does not always involve the same region of the chromosome. Since the start of this study two novel tumour suppressor genes, PTEN (MMAC1) (Li et al, 1997; Steck et al, 1997) and DBMT1 (Mollenhauser et al, 1997) have been identified on the long arm of chromosome 10. One of these, PTEN, has been shown to be mutated in a proportion, but not all, glioma with LOH for chromosome 10 (Rasheed et al, 1997; Bostrom et al, 1998, Fults et al, 1998). Although DBMT1 maps to a region, 10q25–26, which is more frequently associated with LOH in high-grade gliomas (Rasheed et al, 1995), it is also deleted in less than 25% of GBM, suggesting that other significant tumour suppressor genes on chromosome 10 may also be involved in glioma progression. A third candidate tumour suppressor gene, LGI1 (Chernova et al, 1998) has now been identified at 10q24.

Recently Lin et al (1998) have shown that patients with anaplastic astrocytomas or glioblastoma multiforme which show LOH for the region of chromosome 10 around the PTEN (MMAC1) locus have a significantly worse prognosis than patients with no LOH for this region. In their study LOH around the DMBT1 locus had no prognostic significance. In our study, most patients showing LOH for chromosome 10 showed loss of the whole chromosome. In four of the 13 cases with loss of only part of chromosome 10, the marker D10S608 was lost (Figure 4), suggesting that loss of function of PTEN might be involved in these cases. Seven cases showed partial loss involving 10p (Figure 4). In three of these the LOH was confined to markers on 10p. This supports the hypothesis that at least one further tumour suppressor gene may be present on the short arm of chromosome

*© 1999 Cancer Research Campaign* 

10. In cases showing partial LOH involving 10p, all three markers on 10p were lost in one case. Two cases were uninformative with respect to the extent of LOH on 10p. However, four cases showed LOH which was confined to the distal region of chromosome 10, confirming recent data which assigns putative tumour suppressor genes on 10p to the 10p14 and 10p15 regions (Voesten et al, 1997; Ichimura et al, 1998; Kon et al, 1998). Refinement of these regions of loss should lead to the identification of novel tumour suppressor genes involved in glioma progression.

We have shown that LOH for chromosome 10 is an independent prognostic variable in patients with high-grade glioma. Further studies are required to identify novel tumour suppressor genes on chromosome 10 and determine the relative prognostic significance of these and known tumour suppressor genes in patients with high-grade glioma.

### ACKNOWLEDGEMENTS

We gratefully acknowledge funding of this work by the Cancer Treatment and Research Trust, UK (SB, SKN), The Reuter Foundation, UK (SB, SKN) and the Cancer Research Campaign, UK (CB).

### REFERENCES

Barker FG, Davis RL, Chang SM and Prados MD (1996) Necrosis as a prognostic factor in glioblastoma multiforme. *Cancer* **77**: 1161–1166

Bigner SH, Mark J and Bigner DD (1990) Cytogenetics of human brain tumours. *Cancer Genet Cytogenet* **47**: 141–154

Bostrom J, Cobbers JMJL, Wolter M, Tabatabai G, Weber RG, Lichter P, Collins VP and Reifenberger G (1998) Mutation of the PTEN (MMAC1) tumor suppressor gene in a subset of glioblastomas but not in meningiomas with loss of chromosome arm 10q. *Cancer Res* **58**: 29–33

Brada M, Thomas DGT, Bleehen NM, Roberts JT, Senanayake F, Abram P, Lantos PL, Moss TH, Ironside JW, Whaley JB and Stenning SP (1998) Medical Research Council (MRC) randomised trial of adjuvant chemotherapy in high-grade glioma (HGG)-BRO5. *Am Soc Clin Oncol* **17**: 400a

Bruner JM (1994) Neuropathology of malignant gliomas. *Semin Oncol* **21**: 126–138

Campomenosi P, Ottaggio L, Moro F, Urbini S, Bogliolo M, Zunino A, Camoriano A, Inga A, Gentile SL, Pellegata NS, Bonassi S, Bruzzone E, Iannone R, Pisani R, Menichini P, Ranzani GN, Bonatti S, Abbondandolo A and Fronza G (1996) Study on aneuploidy and p53 mutations in astrocytomas. *Cancer Genet Cytogenet* **88**: 95–102

Chang CH, Horton J, Schoenfeld D, Salazer O, Perez-Tamayo R, Kramer S, Weinstein A, Nelson JS and Tsukada Y (1983) Comparison of postoperative radiotherapy and combined postoperative radiotherapy and chemotherapy in the multidisciplinary management of malignant gliomas. A joint Radiation Therapy Oncology Group and Eastern Cooperative Oncology Group study. *Cancer* **52**: 997–1007

Chernova OB, Somerville RP and Cowell JK (1998) A novel gene LGI1, from 10q24 is rearranged and down-regulated in malignant brain tumors. *Oncogene* **17**: 2873–2881

Collins VP (1995) Gene amplification in human gliomas. *Glia* **15**: 289–296

Cox D (1972) Regression models and life-tables. *J R Stat Soc B* **34**: 187–220

Cunningham JM, Kimmel DW, Scheithauer BW, O'Fallon JR, Novotny PJ and Jenkins RB (1997) Analysis of proliferation markers and p53 expression in gliomas of astrocytic origin: relationships and prognostic value. *J Neurosurg* **86**: 121–130

Daumas-Duport C, Scheithauer B, O'Fallon J and Kelly P (1988) Grading of astrocytomas: a simple and reproducible method. *Cancer* **62**: 2152–2165

Fisher RA, Paradinas FJ, Soteriou BA, Foskett M and Newlands ES (1997) Diploid hydatidiform moles with fetal red blood cells in molar villi: 2 – Genetics. *J Pathology* **181**: 189–195

Fults D, Pedone CA, Thompson GE, Uchiyama CM, Gumpper KL, Iliev D, Vinson VL, Tavtigian SV and Perry WL 3rd (1998) Microsatellite deletion mapping on chromosome 10q and mutation analysis of MMAC1, FAS and MXII in human glioblastoma multiforme. *Int J Oncol* **12**: 905–910

Ganju V, Jenkins RB, O'Fallon JR, Scheithauer BW, Ransom DT, Katzmann JA and Kimmel DW (1994) Prognostic factors in gliomas. A multivariate analysis of clinical, pathologic, flow cytometric, cytogenetic, and molecular markers. *Cancer* **74**: 920–927

GDB(™) Human Genome Database [database online]. Baltimore (Maryland, USA): Johns Hopkins University, 1990. Available from Internet: URL http://www.gdb.org

Ichimura K, Schmidt EE, Miyakawa A, Goike HM and Collins VP (1998) Distinct patterns of deletion on 10p and 10q suggest involvement of multiple tumor suppressor genes in the development of astrocytic gliomas of different malignancy grades. *Genes Chromosomes Cancer* **22**: 9–15

Jen J, Harper JW, Bigner SH, Bigner DD, Papadopoulos N, Markowitz S, Willson JK, Kinzler KW and Vogelstein B (1994) Deletion of p15 and p16 genes in brain tumours. *Cancer Res* **54**: 6353–6358

Kaplan E and Meier P (1958) Nonparametric estimation from incomplete observations. *J Am Stat Assoc* **53**: 457–481

Kon H, Sonada Y, Kumaba T, Yoshimoto T, Sekiya T and Murakami Y (1998) Structural and functional evidence for the presence of tumor suppressor genes on the short arm of chromosome 10 in human gliomas. *Oncogene* **16**: 257–263

Korkolopoulou P, Christodoulou P, Kouzelis K, Hadjiyannakis M, Priftis A, Stamoulis, G, Seretis A and Thomas-Tsagli E (1977) MDM2 and p53 expression in gliomas: a multivariate survival analysis including proliferation markers and epidermal growth factor receptor. *Br J Cancer* **75**: 1269–1278

Krouwer HG, Davis RL, Silver P and Prados M (1991) Gemistocytic astrocytomas: a reappraisal. *J Neurosurg* **74**: 399–406

Leenstra S, Oskam NT, Buleveld EH, Bosch DA, Troost D and Hulsebos TJM (1998) Genetic subtype of human malignant astrocytoma correlate with survival. *Int J Cancer* **79**: 159–165

Li J, Yen C, Liaw D, Podsypanina K, Bose S, Wang SI, Puc J, Miliaresis C, Rodgers L, McCombie R, Bigner SH, Giovanella BC, Ittmann M, Tycko B, Hibshoosh H, Wigler MH and Parsons R (1997) PTEN, a putative protein tyrosine phosphatase gene mutated in human, brain, breast and prostate cancer. *Science* **275**: 1943–1947

Lin H, Bondy ML, Langford LA, Hess KR, Delclos GL, Wu X, Chan W, Pershouse MA, Yung WKA and Steck PA (1998) Alleleic deletion analyses of MMAC/PTEN and DMBT1 loci in gliomas: relationship to prognostic significance. *Clin Cancer Res* **4**: 2447–2454

Louis DN and Gusella JF (1995) A tiger behind many doors: multiple genetic pathways to malignant glioma. *Trends Genet* **11**: 412–415

Mollenhauser J, Wiemann S, Scheurlen W, Korn B, Hayashi Y, Wilgenbus KK, von Diemling A and Poustka A (1997) DBMT1, a new member of the SRCR superfamily, on chromosome 10q25.3–26.1 is deleted in malignant brain tumours. *Nature Genet* **17**: 32–39

Newlands ES, O'Reilly SM, Glaser MG, Bower M, Evans H, Brock C, Brampton MH, Colquhoun I, Lewis P, Rice-Edwards JM, Illingworth RD and Richards PG (1996) The Charing Cross Hospital experience with temozolomide in patients with gliomas. *Eur J Cancer* **32A**: 2236–2241

Peto R, Pike M, Armitage P, Breslow N, Cox D, Howard S, Mantel N, McPhearson K, Peto J and Smith P (1977) Design and analysis of randomised clinical trials requiring prolonged observation of each patient II: analysis and examples. *Br J Cancer* **35**: 1–39

Rainov NG, Dobberstein KU, Bahn H, Holzhausen HJ, Lautenschlager C, Heidecke V and Burkert W (1997) Prognostic factors in malignant glioma: influence of the overexpression of oncogene and tumor-suppressor gene products on survival. *J Neurooncol* **35**: 13–28

Rasheed BK, McLendon RE, Friedman HS, Friedman AH, Fuchs HE, Bigner DD and Bigner SH (1995) Chromosome 10 deletion mapping in human gliomas: a common deletion in 10q25. *Oncogene* **10**: 2243–2246

Rasheed BKA, Stenzel TT, McLendon RE, Parsons R, Friedman AH, Friedman HS, Bigner DD and Bigner SH (1997) PTEN gene mutations are seen in high-grade but not in low-grade gliomas. *Cancer Res* **57**: 4187–4190

Report of the Medical Research Council Brain Tumour Working Party (1990) Prognostic factors for high-grade malignant glioma: development of a prognostic index. *J Neurooncol* **9**: 47–55

Steck PA, Pershouse MA, Jasser SA, Yung WKA, Lin H, Ligon AH, Langford LA, Baumgard ML, Hattier T, Davis T, Frye C, Hu R, Swedlund B, Teng DHF and Tavtigian SV (1997) Identification of a candidate tumour suppressor gene MMAC1, at chromosome 10q23.3 that is mutated in multiple advanced cancers. *Nature Genet* **15**: 356–362

Voesten AM, Bijleveld EH, Westerveld A and Hulsebos TJ (1997) Fine mapping of a region of common deletion on chromosome arm 10p in human glioma. *Genes Chromosomes Cancer* **20**: 167–172

von Deimling A, Louis DN and Wiestler OD (1995) Molecular pathways in the formation of gliomas. *Glia* **15**: 328–338

 © 1999 Cancer Research Campaign

Waha A, Baumann A, Wolf HK, Fimmers R, Neumann J, Kindermann D, Astrahantseff K, Blumcke I, von-Deimling A and Schlegel U (1996) Lack of prognostic relevance of alterations in the epidermal growth factor receptor-transforming growth factor-alpha pathway in human astrocytic gliomas. *J Neurosurg* **85**: 634–641

Wright DK and Manos MM (1990) Sample preparation from paraffin-embedded tissues. In: *PCR Protocols, a Guide to Methods and Applications*, Innis MA, Gelfand DH, Sninsky JJ and White TJ (eds), pp. 153–158. Academic Press: London

Zhu A, Shaeffer J, Leslie S, Kolm P and El-Mahdi AM (1996) Epidermal growth factor receptor: an independent predictor of survival in astrocytic tumors given definitive irradiation. *Int J Radiation Oncol Biol Phys* **34**: 809–815

© 1999 Cancer Research Campaign
